# Nonsense and Sense Suppression Abilities of Original and Derivative *Methanosarcina mazei* Pyrrolysyl-tRNA Synthetase-tRNA^Pyl^ Pairs in the *Escherichia coli* BL21(DE3) Cell Strain

**DOI:** 10.1371/journal.pone.0057035

**Published:** 2013-03-08

**Authors:** Keturah A. Odoi, Ying Huang, Yohannes H. Rezenom, Wenshe R. Liu

**Affiliations:** Department of Chemistry, Texas A&M University, College Station, Texas, United States of America; Max-Planck-Institute for Terrestrial Microbiology, Germany

## Abstract

Systematic studies of nonsense and sense suppression of the original and three derivative *Methanosarcina mazei* PylRS-tRNA^Pyl^ pairs and cross recognition between nonsense codons and various tRNA^Pyl^ anticodons in the *Escherichia coli* BL21(DE3) cell strain are reported. 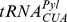
is orthogonal in *E. coli* and able to induce strong amber suppression when it is co-expressed with pyrrolysyl-tRNA synthetase (PylRS) and charged with a PylRS substrate, *N^ε^*-tert-butoxycarbonyl-l-lysine (BocK). Similar to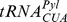, 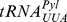
is also orthogonal in *E. coli* and can be coupled with PylRS to genetically incorporate BocK at an ochre mutation site. Although 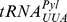
is expected to recognize a UAG codon based on the wobble hypothesis, the PylRS-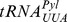 pair does not give rise to amber suppression that surpasses the basal amber suppression level in *E. coli*. *E. coli* itself displays a relatively high opal suppression level and tryptophan (Trp) is incorporated at an opal mutation site. Although the PylRS-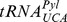 pair can be used to encode BocK at an opal codon, the pair fails to suppress the incorporation of Trp at the same site. 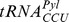 fails to deliver BocK at an AGG codon when co-expressed with PylRS in *E. coli*.

## Introduction

**Figure 1:**
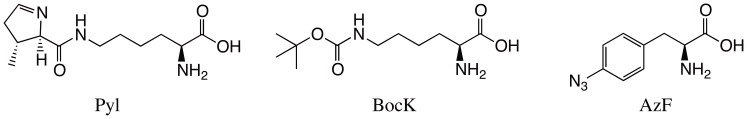
The structures of Pyl, BocK and AzF.

Pyrrolysine (Pyl, **Figure 1**), the 22^nd^ proteinogenic amino acid that was originally discovered in methanogenic methylamine methyltransferase, is genetically encoded by the RNA nucleotide triplet UAG, a stop codon that halts translation of mRNA during a regular protein translation process [1]. The delivery of Pyl to ribosome is mediated by a unique tRNA, 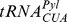
that is specifically aminoacylated by a unique aminoacyl-tRNA synthetase (aaRS), pyrrolysyl-tRNA synthetase (PylRS) [2]. 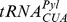
contains a special nucleotide triplet CUA, an anticodon that recognizes a UAG stop codon in mRNA. Unlike tRNA^Sec^ that needs a special elongation factor (SelB in *E. coli* and EFsec in mammalian cells) and an mRNA secondary structure for its binding to the ribosome A site and recognition of a UGA stop codon for the delivery of selenocysteine, 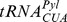 hijacks the regular translation elongation process to suppress a UAG codon for the incorporation of Pyl [3]–[5]. Previous studies also demonstrated that PylRS shows remarkably high substrate promiscuity and is able to charge 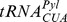 with a variety of noncanonical amino acids (NAAs). For these reasons and due to the naturally high orthogonality of the PylRS-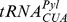 pair in bacteria, yeast, and mammalian cells, this pair has been directly transferred to *E. coli*, *Saccharomyces cerevisiae*, and human cells for the genetic incorporation of more than ten lysine derivatives including *N^ε^*-tert-butoxycarbonyl-l-lysine (BocK) into proteins at amber mutation sites[6]–[12]. Engineered PylRS-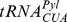 pairs have also been used to genetically encode other lysine derivatives and even phenylalanine derivatives that are structurally distinctive from Pyl[13]–[21]. The genetic incorporation of these NAAs into proteins and their following modifications allow a variety of biochemistry studies such as the functional investigation of protein posttranslational modifications, protein folding dynamic analysis, biosensor development, tracking signal transduction processes, and probing enzyme mechanisms [22]. Pioneered by Schultz and coworkers on the incorporation of two different NAAs [23], two methods were recently developed independently in the Chin Group and our group that couple the wild-type or a derivative PylRS-tRNA^Pyl^ pair with another suppressing aaRS-tRNA pair for the genetic incorporation of two different NAAs into one protein in *E. coli* [24], [25]. The method developed in the Chin Group used one amber codon and one quadruple AGGA codon that were suppressed by the PylRS-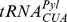 pair and an evolved *M. jannaschii* tyrosyl-tRNA synthetase *Mj*TyrRS-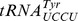 pair, respectively to code two different NAAs. A specially engineered ribosome, Ribo-Q1, was used to improve the AGGA suppression level. Our method relied on the suppression of two stop codons, namely one amber UAG codon and one ochre UAA codon, which was achieved by genetically encoding an evolved *Mj*TyrRS-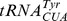 pair for amber suppression and a wild type or evolved PylRS-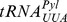 pair for ochre suppression in *E. coli*. The genetic incorporation of two different NAAs into one protein can be potentially applied to install a FRET pair in a protein for conformation and dynamic studies as we demonstrated in a separate publication [26], synthesize proteins with two different posttranslational modifications for their functional investigation, and build phage-displayed peptide libraries with the expanded chemical diversities.

Although the PylRS-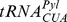 pair has been used extensively for the genetic incorporation of different NAAs in the past few years, two questions related to the pair have not been fully addressed. While our lab and other groups have showed that mutating the anticodon of 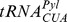
does not significantly affect its interaction with the catalytic domain of PylRS [27], further investigation need to be done to determine whether we can directly use mutant tRNA^Pyl^ forms for the genetic incorporation of NAAs at an opal or ochre codon or even a sense codon in *E. coli*. Another study is necessary to clarify whether an aminoacylated 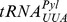
can lead to amber suppression since a UUA anticodon can recognize a UAG codon based on the wobble hypothesis [28]. In this study, we attempted to address these two questions and carried out all the experiments in the *E. coli* BL21(DE3) cell strain which has been broadly used for the genetic incorporation of NAAs.

## Materials and Methods

### Materials

Phusion high-fidelity DNA polymerase, T4 DNA ligase, T4 polynucleotide kinase, and restriction enzymes were purchased from New England Biolabs. Oligonucleotide primers were ordered from Integrated DNA Technologies. Ni-NTA superflow resins were purchased from Qiagen. All polymerase chain reactions (PCRs) were performed using Phusion high-fidelity DNA polymerase. BocK was purchased from Chem Impex. *p*-Azido-l-phenylalanine (AzF) was synthesized according to a revised literature procedure [29].

### Plasmids

Plasmid pETtrio-pylT(UUA)-PylRS-MCS was derived from pPylRS-pylT-GFP1TAG149TAA [25] and carries a 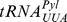 gene (a C34U mutant form of 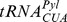) under control of the *lpp* promoter and the *rrnC* terminator, the wild type *Methanosarcina mazei* PylRS gene under control of the *glnS* promoter and terminator, and multiple cloning sites including *NcoI, NotI, SalI and KpnI* targeted sites under control of the T7 promoter and terminator. Plasmid pETtrio-pylT(UUA)-PylRS-sfGFP134TAG that carries an additional superfolder green fluorescent protein (sfGFP) gene with an amber mutation at N134 (sfGFP134TAG) was constructed by cloning the sfGFP134TAG gene to the *NcoI* and *KpnI* sites of pETtrio-pylT(UUA)-PylRS-MCS.

Three plasmids pETtrio-pylT(CUA)-PylRS-sfGFP134TAG, pETtrio-pylT(UUA)-PylRS-sfGFP134TAA, and pETtrio-pylT(UCA)-PylRS-sfGFP134TGA that vary at the anticodon of tRNA^Pyl^ and have different nonsense mutations at N134 of the sfGFP gene were derived from pETtrio-pylT(UUA)-PylRS-sfGFP134TAG. Constructions of these plasmids were carried out using a site-directed mutagenesis protocol that was based on Phusion DNA polymerase. In brief, two oligonucleotide primers, one of which covers the mutation site were used to amplify the whole plasmid of pETtrio-pylT(UUA)-PylRS-sfGFP134TAG to give a blunt-end PCR product. This PCR product was phosphorylated by T4 polynucleotide kinase and then self-ligated using T4 DNA ligase. Plasmid pETtrio-pylT(CUA)-PylRS-sfGFP134TAG carries genes coding 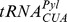, PylRS, and sfGFP134TAG; plasmid pETtrio-pylT(UUA)-PylRS-sfGFP134TAA carries genes coding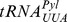, PylRS, and sfGFP with an ochre mutation at N134 (sfGFP134TAA); and plasmid pETtrio-pylT(UCA)-PylRS-sfGFP134TGA carries genes coding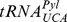, PylRS, and sfGFP with an opal mutation at N134 (sfGFP134TGA). Plasmid pETtrio-pylT(CUA)-PylRS-sfGFP134TAA that carries genes coding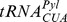, PylRS, and sfGFP134TAA was derived from plasmid pETtrio-pylT(UUA)-PylRS-sfGFP134TAA.

Plasmid pETtrio-sfGFP134TGA was derived from pETtrio-pylT(UCA)-PylRS-sfGFP134TGA by digesting it with *SphI* to remove the 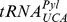 gene and parts of the PylRS gene and self-ligating the purified digested plasmid backbone. Plasmid pETtrio-pylT(UCA)-PylRS-sfGFP2TGA carries genes coding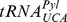, PylRS, and sfGFP with an opal mutation at S2 (sfGFP2TGA). To construct this plasmid, the sfGFP2TGA gene was used to replace the sfGFP134TGA gene in pETtrio-pylT(UCA)-PylRS-sfGFP134TGA. Plasmid pETtrio-pylT(CCU)-PylRS-sfGFP2AGG was constructed from pETtrio-pylT(CUA)-PylRS-sfGFP2TGA using two consecutive Phusion DNA polymerase-based site directed mutagenesis steps.

Three plasmids pETtrio-pylT(UCA)G73C-PylRS-sfGFP134TGA, pETtrio-pylT(UCA)G73U-PylRS-sfGFP134TGA, and pETtrio-pylT(UCA)G73A-PylRS-sfGFP134TGA carry mutations that change G73 of 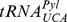
to C, U, and A, respectively. These plasmids were derived from pETtrio-pylT(UCA)-PylRS-sfGFP134TGA using Phusion DNA polymerase-based site-directed mutagenesis.

### Amber, Opal, and Ochre Suppression

Plasmids pETtrio-pylT(CUA)-PylRS-sfGFP134TAG, pETtrio-pylT(UCA)-PylRS-sfGFP134TGA, and pETtrio-pylT(UUA)-PylRS-sfGFP134TAA were individually used to transform *E. coli* BL21(DE3) cells. For each plasmid, a single colony was selected and allowed to grow in 5 mL of LB medium with 100 µg/mL ampicillin at 37°C overnight. The overnight culture was inoculated into 200 mL of 2YT medium with 100 µg/mL ampicillin and allowed to grow at 37°C to OD_600_∼1.2. 1 mM isopropyl-β-D-thiogalactopyranoside (IPTG) and 5 mM BocK were then added to the medium to induce expression of sfGFP. Control experiments in which only 1 mM IPTG was added to the medium were also carried out. The induced cells were allowed to grow at 37°C overnight and then collected by centrifugation (4,200 rpm for 20 min). The collected cells were resuspended in 35 mL of lysis buffer (50 mM HEPES, 300 mM NaCl, 10 mM imidazole, pH 8.0) and lysed by sonication in an ice water bath. The lysed cells were clarified by centrifugation (10,000 rpm for 1 h). The supernatant was decanted and let bind to 5 mL of Ni-NTA superflow resins at 4°C for 1 h. The mixture of the supernatant and resins was then loaded to an empty Qiagen Ni-NTA superflow cartridge. The resins were washed with 5 volume times of lysis buffer and sfGFP was then eluted with buffer (50 mM HEPES, 300 mM NaCl, 250 mM imidazole, pH 8.0). To further purify the expressed sfGFP, the protein was equilibrated against buffer A (20 mM Bis-Tris, pH 6.1) and then loaded to a monoS column from GE Health Science. The protein was eluted out by running a gradient from buffer A to 100% of buffer B (20 mM Bis-Tris, 1 mM NaCl, pH 6.1). The finally purified protein was then concentrated to a desired volume and analyzed by SDS-PAGE. To analyze the purified protein by electrospray ionization mass spectrometry (ESI-MS) analysis, the buffer of the purified protein was changed to the phosphate buffer saline. Without further indication, protein purification and characterization in the following experiments were the same.

### Suppression of an Opal Mutation at S2 of sfGFP

Plasmid pETtrio-pylT(UCA)-PylRS-sfGFP2TGA was used to transform *E. coli* BL21(DE3) cells. A single colony was then used to express sfGFP at two induction conditions: (1) 1 mM IPTG and (2) 1 mM IPTG and 5 mM BocK.

### Basal Suppression at an Opal UGA Codon

Plasmid pETtrio-sfGFP134TGA was used to transform *E. coli* BL21(DE3) cells. A single colony was then selected to do protein expression that was induced by the addition of 1 mM IPTG.

### Mutagenic Analysis of 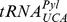


Plasmids pETtrio-pylT(UCA)G73C-PylRS-sfGFP134TGA, pETtrio-pylT(UCA)G73U-PylRS-sfGFP134TGA, and pETtrio-pylT(UCA)G73A-PylRS-sfGFP134TGA were used individually to transform *E. coli* BL21(DE3) cells. A single colony for each plasmid was then selected to do protein expression at two induction conditions: (1) 1 mM IPTG and (2) 1 mM IPTG and 5 mM BocK.

### Anticodon-codon Cross Recognition

Plasmids pETtrio-pylT(CUA)-PylRS-sfGFP134TAG, pETtrio-pylT(CUA)-PylRS-sfGFP134TAA, pETtrio-pylT(UUA)-PylRS-sfGFP134TAG, and pETtrio-pylT(UUA)-PylRS-sfGFP134TAA were individually used to transform *E. coli* BL21(DE3) cells. A single colony for each plasmid was then selected and allowed to grow in 5 mL of LB medium with 100 µg/mL ampicillin at 37°C overnight. This overnight culture was then inoculated into 500 mL of 2YT medium with 100 µg/mL ampicillin and allowed to grow to OD_600_∼1.2. 1 mM IPTG and 5 mM BocK were then provided and cells were allowed to grow at 37°C overnight. For pETtrio-pylT(UUA)-PylRS-sfGFP134TAG, expression of sfGFP in the absence of BocK was also tested.

### Competitive Recognition of the Third Nucleotide of an Amber Codon

Plasmid pEVOL-AzFRS was a gift from Dr. Peter Schultz at Scripps Research Institute [30]. It carries one 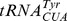 gene under control of a *proK* promoter and a *proK* terminator, an evolved AzF-specific *Mj*TyrRS (AzFRS) gene under control of a *glnS* promoter and a *glnS* terminator, and an AzFRS gene under control of a pBAD promoter. This plasmid together with pETtrio-pylT(UUA)-PylRS-sfGFP134TAG was used to co-transform *E. coli* BL21(DE3) cells. One single colony was selected and allowed to grow in 5 mL of LB medium with 100 µg/mL ampicillin and 34 µg/mL chloramphenicol at 37°C overnight. This overnight culture was inoculated into 200 mL of 2YT medium with 100 µg/mL ampicillin and 34 µg/mL chloramphenicol and allowed to grow to OD_600_∼1.2. Expression of sfGFP was then induced. Four induction conditions were tested, including (1) 1 mM IPTG only, (2) 1 mM IPTG and 1 mM AzF, (3) 1 mM IPTG and 5 mM BocK, and (4) 1 mM IPTG, 1 mM AzF, and 5 mM BocK.

### AGG Codon Suppression

Plasmid pETtrio-pylT(CCU)-PylRS-sfGFP2AGG was used to transform *E. coli* BL21(DE3) cells. A single colony was then selected and allowed to grow in 5 mL of LB medium with 100 µg/mL ampicillin at 37°C overnight. This overnight culture was then inoculated into 500 mL of 2YT medium with 100 µg/mL ampicillin and allowed to grow to OD_600_∼1.2. Two conditions were used to induce sfGFP expression. One is the addition of 1 mM IPTG and the other is the addition of 1 mM IPTG and 5 mM BocK.

## Results

### Amber, Opal, and Ochre Suppression Efficiencies of the PylRS-tRNA^Pyl^ Pairs

**Figure 2:**
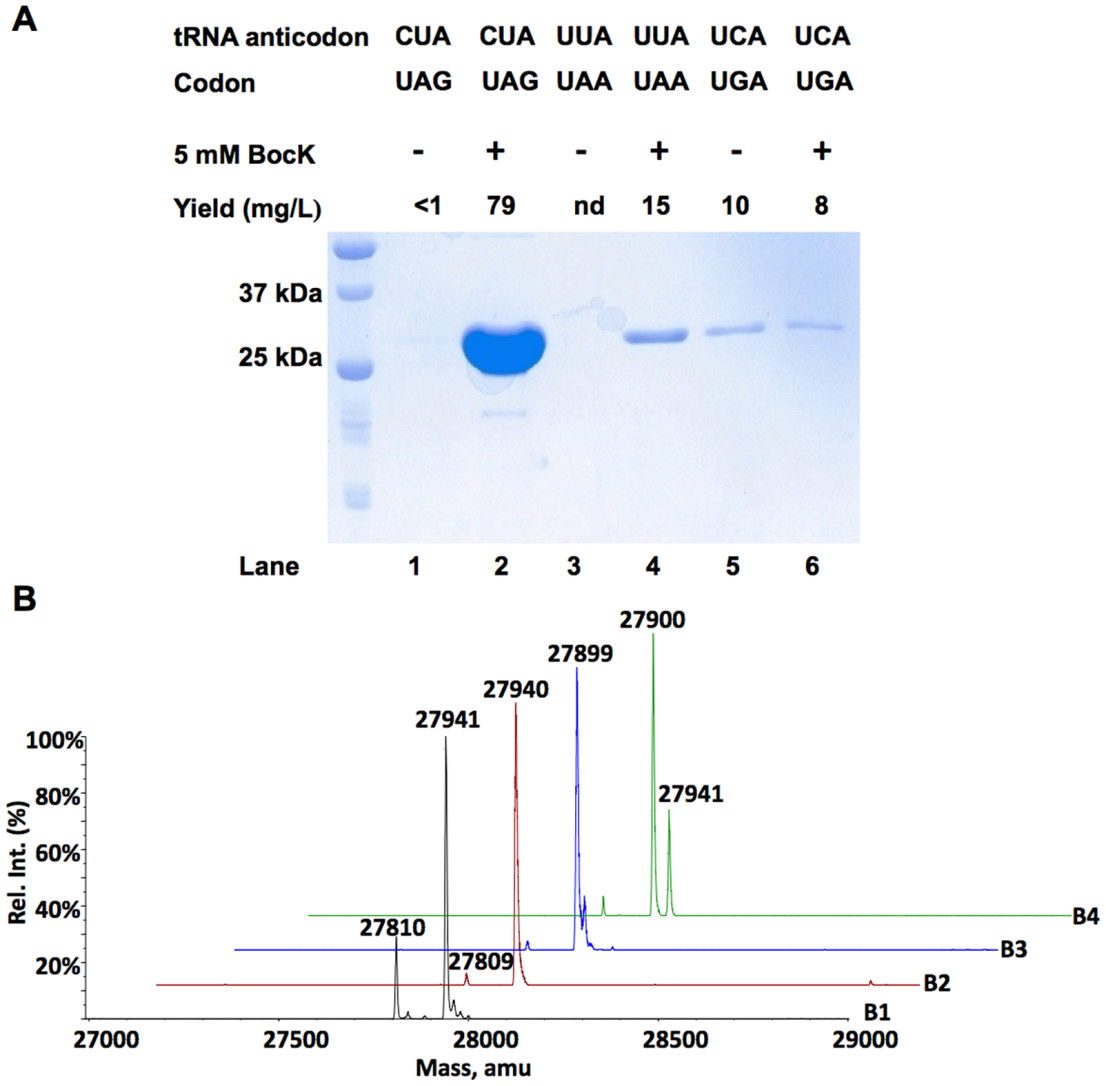
Suppression of amber, opal, and ochre mutations at N134 of sfGFP by their corresponding PylRS-tRNA^Pyl^ pairs in the absence and presence of BocK. (**A**) Proteins shown in the gel represent their real relative sfGFP expression levels. Lanes 1 and 2 were transformed with pETtrio-PylT(CUA)-PylRS-sfGFP134TAG; lanes 3 and 4 were transformed with pETtrio-PylT(UUA)-PylRS-sfGFP134TAA; lanes 5 and 6 were transformed with pETtrio-PylT(UCA)-PylRS-sfGFP134TGA. ESI-MS spectra of sfGFP expressed in cells (**B1**) transformed with pETtrio-PylT(CUA)-PylRS-sfGFP134TAG and grown in the presence of 5 mM BocK, (**B2**) transformed with pETtrio-PylT(UUA)-PylRS-sfGFP134TAA and grown in the presence of 5 mM BocK, (**B3**) transformed with pETtrio-PylT(UCA)-PylRS-sfGFP134TGA and grown in the absence of BocK, and (**B4**) transformed with pETtrio-PylT(UCA)-PylRS-sfGFP134TGA and grown in the presence of 5 mM BocK.

To demonstrate amber suppression efficiency of the PylRS-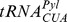 pair, *E. coli* BL21(DE3) cells transformed with pETtrio-pylT(CUA)-PylRS-sfGFP134TAG were used to express sfGFP both in the absence and in the presence of 5 mM BocK, a substrate of PylRS. Without BocK in the growth medium, low level of sfGFP expression was observed. On the contrary, the addition of BocK promoted sfGFP overexpression (**Figure 2A**). The ESI-MS analysis of the purified sfGFP displayed two major mass peaks at 27,810±1 Da and 27,941±1Da that agree well with the theoretical molecular weights of sfGFP with BocK incorporated at N134 (27,940 Da for the full-length protein; 27809 Da for the full-length protein without the first methionine (M1)) (**Figure 2B** **1**). *E. coli* BL21(DE3) cells transformed with pETtrio-pylT(UUA)-PylRS-sfGFP134TAA showed an undetectable expression level of sfGFP when BocK was absent in the growth medium. The addition of BocK induced sfGFP expression (**Figure 2A**). The ESI-MS analysis of the purified sfGFP showed two mass peaks (27,940±1 Da and 27,809±1 Da) that agree well with the theoretical molecular weights of sfGFP with BocK incorporated at N134 (**Figure 2B** **2**). In comparison to amber suppression, the expression level of sfGFP using ochre suppression is lower. *E. coli* BL21(DE3) cells transformed with pETtrio-pylT(UCA)-PylRS-sfGFP134TGA exhibited a high expression level of sfGFP both in the absence and in the presence of BocK in the growth medium (**Figure 2A**). The ESI-MS analysis of purified sfGFP expressed in the absence of BocK showed a mass peak at 27,899±1 Da that clearly matched a Trp residue at N134 of sfGFP (calculated mass: 27,898 Da) (**Figure 2B** **3**). The ESI-MS analysis of sfGFP expressed in the presence of BocK displayed a very interesting spectrum. Mass peaks for both Trp residue at N134 of sfGFP (27,900±1 Da) and BocK residue at N134 of sfGFP (27,941±1 Da) were observed. The mass peak for the Trp isoform was much more intensive than the BocK isoform (**Figure 2B** **4**).

**Figure 3:**
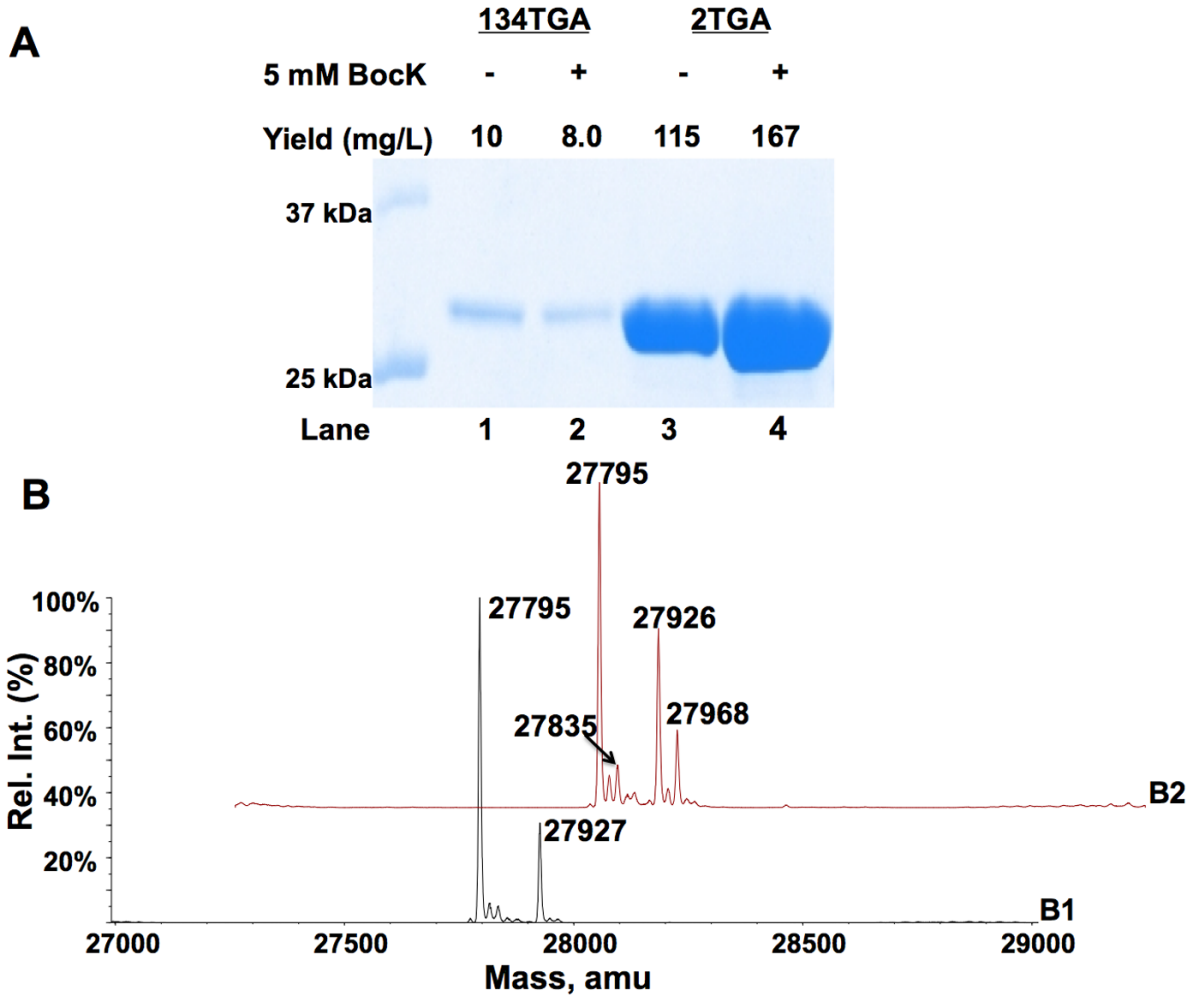
Suppression of an opal mutation at S2 of sfGFP by the PylRS-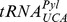 pair. (**A**) Expression of sfGFP with an opal mutation. Lanes 1 and 2 were transformed with pETtrio-pylT(UCA)-sfGFP134TGA and grown in the absence or presence of 5 mM BocK; lanes 3 and 4 were transformed with pETtrio-pylT(UCA)-sfGFP2TGA and grown in the absence or presence of 5 mM BocK. Each protein shown in the gel represents their real relative expression levels. ESI-MS of sfGFP expressed in cells transformed with pETtrio-pylT(UCA)-sfGFP2TGA and grown in the (**B1**) absence or (**B2**) presence of 5 mM BocK.

**Figure 4:**
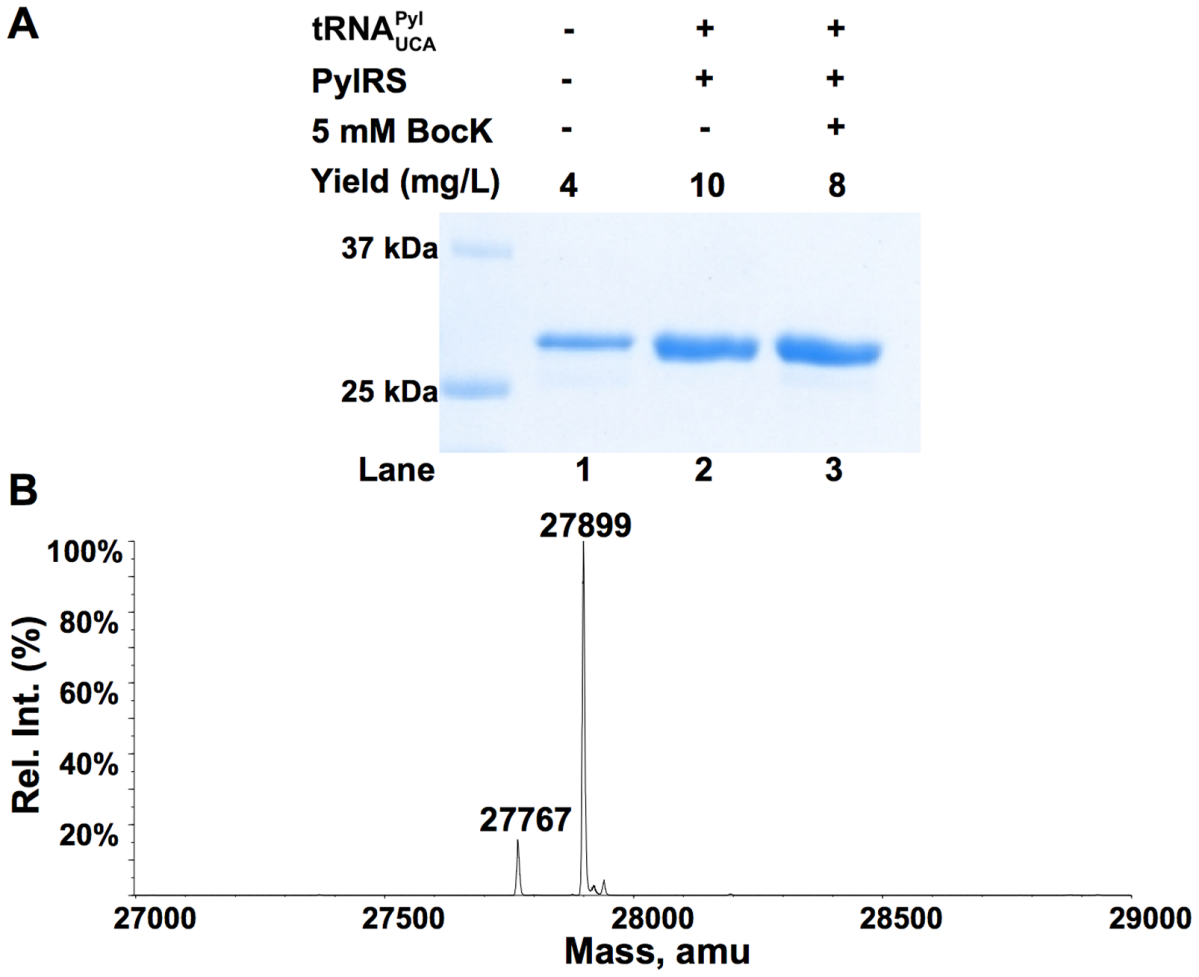
Suppression of an opal mutation at N134 of sfGFP at different conditions. (**A**) Proteins shown in the gel represent their real relative expression levels. Lane 1 was transformed with pET-sfGFP134TGA; lanes 2 and 3 were transformed with pET-pylT(UCA)-sfGFP134TGA and grown in the absence or presence of 5 mM BocK. (**B**) ESI-MS of sfGFP expressed in cells transformed with pETtrio-sfGFP134TGA.

To test whether the incorporation of Trp at the 134 position is related to the nucleotide contents of mRNA around the UGA codon, plasmid pETtrio-pylT(UCA)-PylRS-sfGFP2TGA was constructed to test the opal suppression. *E. coli* BL21(DE3) cells transformed with pETtrio-pylT(UCA)-PylRS-sfGFP2TGA displayed high sfGFP expression levels both in the absence and in the presence of BocK that were much higher than in cells transformed with plasmid pETtrio-pylT(UCA)-PylRS-sfGFP134TGA and grown in the same conditions (**Figure 3A**). The ESI-MS analyses of purified sfGFP from cells transformed with pETtrio-pylT(UCA)-PylRS-sfGFP2TGA displayed similar patterns as sfGFP from cells transformed with pETtrio-pylT(UCA)-PylRS-sfGFP134TGA. Two mass peaks for sfGFP expressed both in the absence and in the presence of BocK (27,795±1 Da and 27,927±1 Da in **Figure 3B** **1** and 27,795±1 Da and 27,926±1 Da in **Figure 3B** **2**) match the molecular weights of sfGFP with Trp incorporated at S2 (calculated mass: 27,926 Da for the full-length protein; 27,794 Da for the full-length protein without M1). For sfGFP expressed in the presence of 5 mM BocK, there were two small peaks at 27,968±1 Da and 27,835±1 Da that agree well with the molecular weights of sfGFP with BocK incorporated at S2 (calculated mass: 27,967 Da for the full-length protein; 27,836 Da for the full-length protein without M1). To further understand opal suppression in the *E. coli* BL21(DE3) cell strain, plasmid pETtrio-sfGFP134TGA that carries no opal suppressing 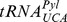
was used to transform *E. coli* BL21(DE3) cells. The transformed cells displayed a relatively high detectable level of sfGFP expression with a yield of 4 mg/L (**Figure 4A**).The ESI-MS analysis of sfGFP resulted from this basal opal suppression showed major mass peaks at 27,899±1 Da and 27,767±1 Da (**Figure 4B**). They match the molecular weights of sfGFP with Trp incorporated at N134.

**Figure 5:**
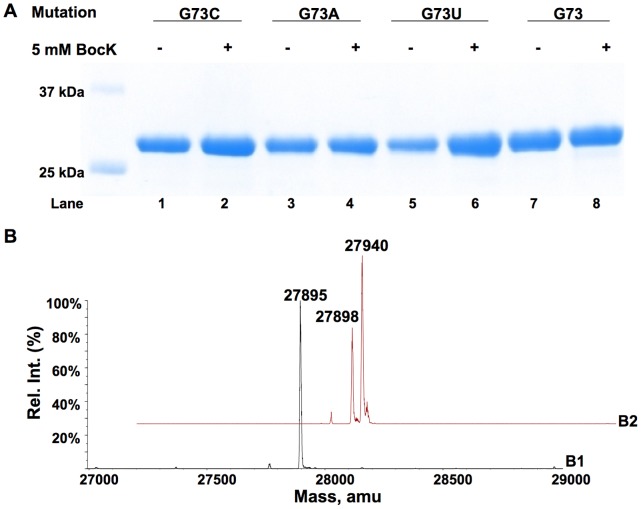
Suppression of an opal mutation at N134 of sfGFP by different 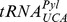 variants. (**A**) Proteins shown in the gel represent their real relative expression levels. Lanes 1 and 2 were transformed with pETtrio-pylT(UCA)G73C-sfGFP134TGA and grown in the absence or presence of 5 mM BocK; lanes 3 and 4 were transformed with pETtrio-pylT(UCA)G73A-sfGFP134TGA and grown in the absence or presence of 5 mM BocK; lanes 5 and 6 were transformed with pETtrio-pylT(UCA)G73U-sfGFP134TGA and grown in the absence or presence of 5 mM BocK; lanes 7 and 8 were transformed with pETtrio-pylT(UCA)-sfGFP134TGA and grown in the absence or presence of 5 mM BocK. The ESI-MS analysis of sfGFP expressed in cells transformed with pETtrio-pylT(UCA)G73U-sfGFP134TGA and grown in the (**B1**) absence or (**B2**) presence of 5 mM BocK.

The suppression efficiencies of three mutated forms of 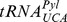
with mutations as G73A, G73C, and G73U, respectively were also examined. Cells transformed with either pETtrio-pylT(UCA)G73A-PylRS-sfGFP134TGA or pETtrio-pylT(UCA)G73C-PylRS-sfGFP134TGA displayed similar expression levels of sfGFP both in the presence and in the absence of BocK(**Figure 5A**). However, cells transformed with pETtrio-pylT(UCA)G73U-PylRS-sfGFP134TGA showed significantly different expression levels of sfGFP when grown in the absence and in the presence of BocK. The ESI-MS analysis of sfGFP expressed in the absence of BocK showed a major mass peak at 27,895±1 Da that matches sfGFP with Trp incorporated at N134 (**Figure 5B** **1**). The addition of 5 mM BocK promoted the sfGFP expression level to increase. The ESI-MS analysis of the purified protein confirmed sfGFP with BocK incorporated at N134 became dominant (**Figure 5B** **2**). The intensity of the mass peak at 27,940±1 Da that matches sfGFP with BocK incorporated at N134 is roughly twice of the mass peak at 27,898±1 Da that matches sfGFP with Trp incorporated at N134.

### Anticodon-codon Cross Recognition

**Figure 6:**
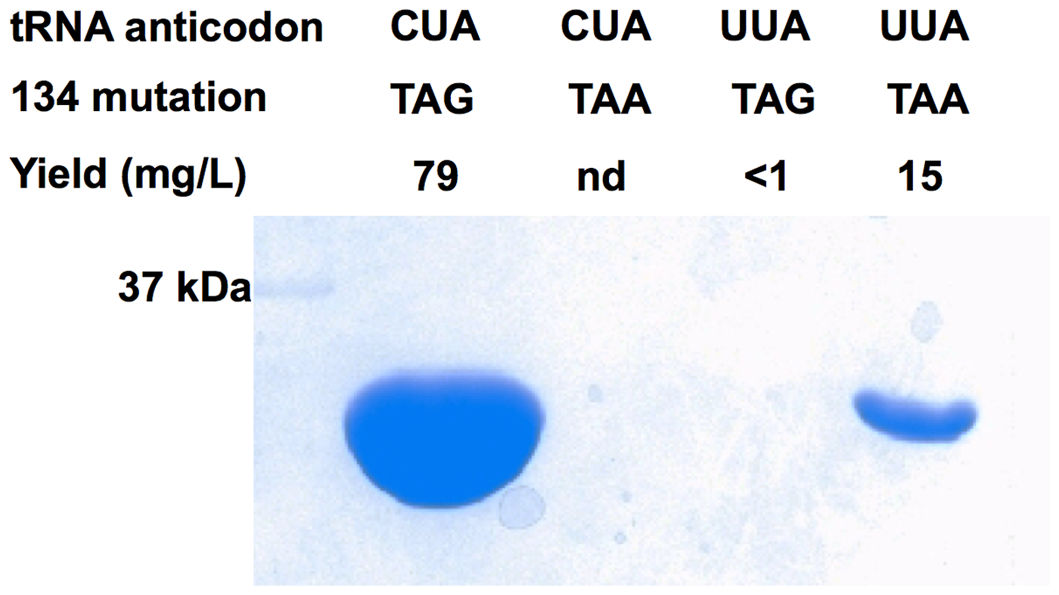
Cross recognitions between different anticodons of tRNA^Pyl^ and nonsense mutations at N134 of sfGFP. Cells were transformed with pETtrio-PylT(NNN)-PylRS-sfGFP134N’N’N’ and grown in the presence of 5 mM BocK (NNN and N’N’N’ denote anticodons and codons specified in the figure). Proteins shown in the gel represent their real relative expression levels.

**Figure 7:**
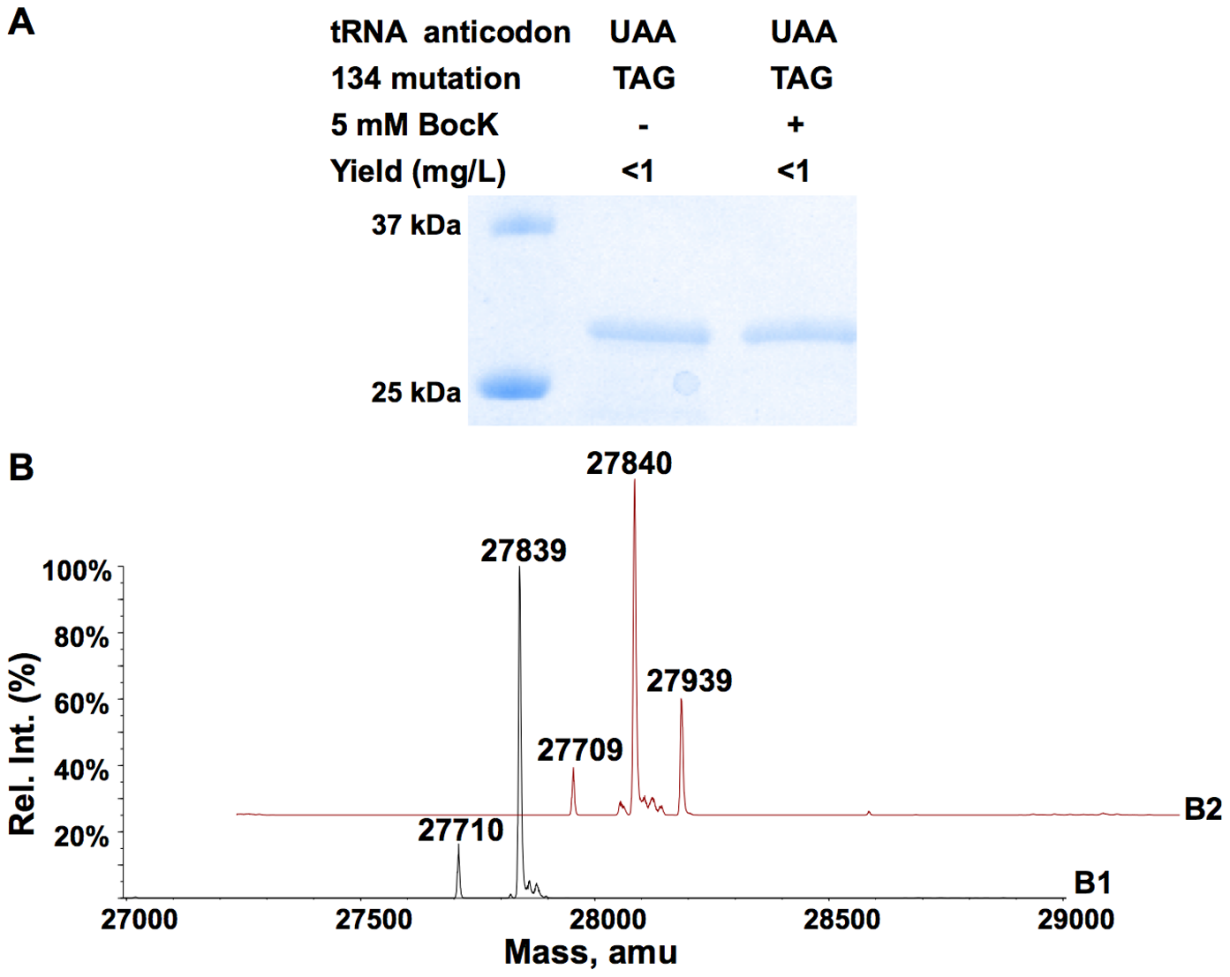
Expression of sfGFP in cells transformed with pETtrio-PylT(UUA)-PylRS-sfGFP134TAG. (**A**) Cells grown in 2YT medium supplemented without or with BocK. ESI-MS of sfGFP expressed in the (**B1**) absence or (**B2**) presence of 5 mM BocK.

Transforming *E. coli* BL21(DE3) cells with pETtrio-pylT(CUA)-PylRS-sfGFP134TAA followed by growing cells in the presence of 5 mM BocK did not lead to detectable expression of sfGFP. *E. coli* BL21(DE3) cells transformed with pETtrio-pylT(UUA)-PylRS-sfGFP134TAG showed a detectable but low level of sfGFP expression (less than 1 mg/L) (**Figure 6**). To see whether BocK promoted suppression at the amber mutation site, *E. coli* BL21(DE3) cells transformed with pETtrio-pylT(UUA)-PylRS-sfGFP134TAG was also grown in the absence of BocK. As shown in **Figure 7A**, the sfGFP expression levels both in the absence and in the presence of BocK were very similar. Addition of BocK did not lead to significant increase of amber suppression. As shown in **Figures 7B** **1**&**B2**, sfGFP expressed in both conditions displayed mass peaks (27,839±1 Da and 27,710±1 Da in **Figure 7B** **1** and 27,840±1 Da and 27,709±1 Da in **Figure 7B** **2**) that match sfGFP with glutamic acid (Glu), lysine (Lys), or glutamine(Gln) incorporated at N134. **Figure 7B** **2** did show a mass peak at 27,939±1 Da that matches the molecular weight of sfGFP with BocK incorporated at N134. However, its intensity was much lower than the mass peak of sfGFP at ∼27,840±1 Da.

**Figure 8:**
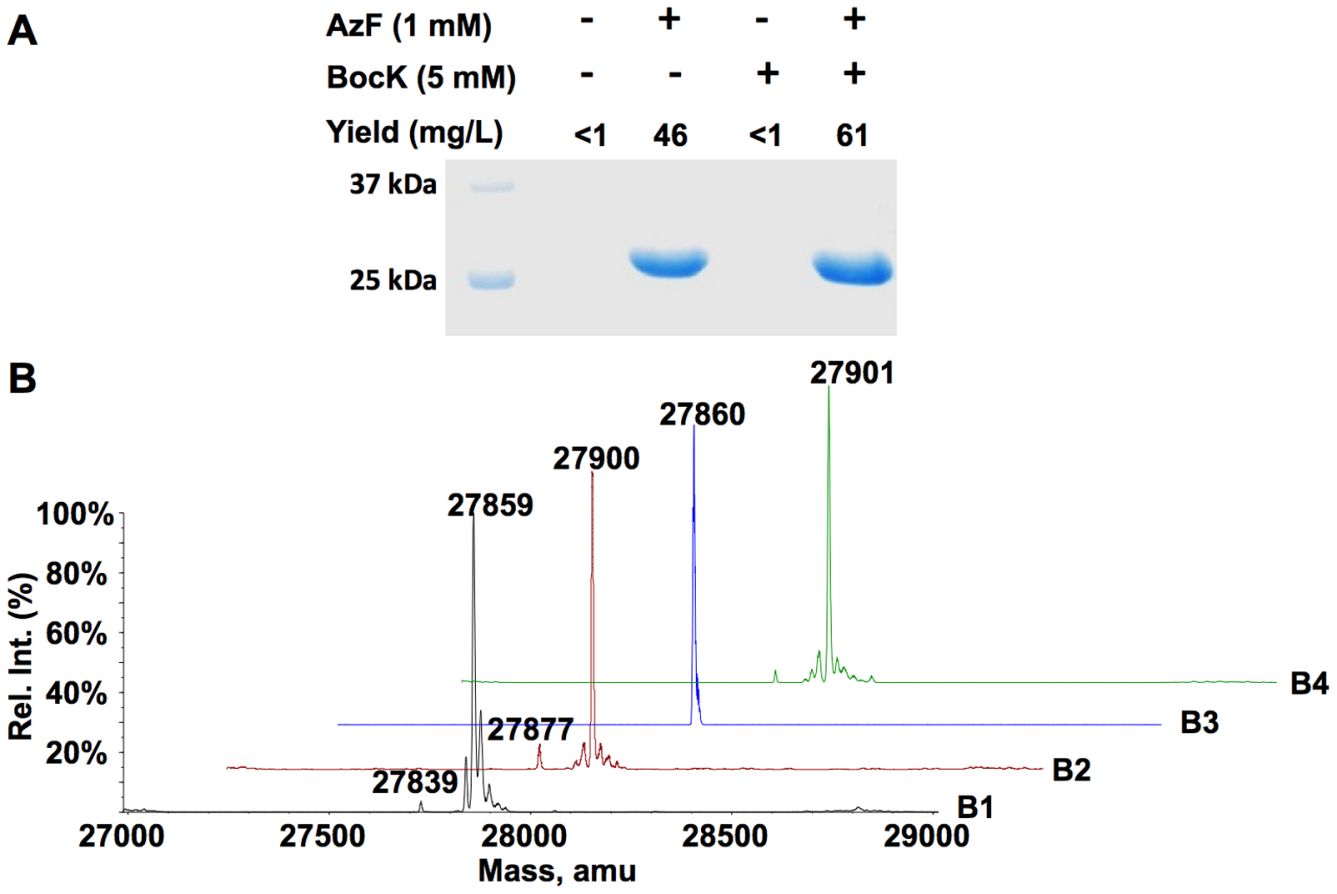
Expression of sfGFP in cells transformed with pETtrio-PylT(UUA)-PylRS-sfGFP134TAG and pEVOL-AzFRS. (**A**)Cells were grown in 2YT medium supplemented with different combinations of NAAs. ESI-MS of sfGFP expressed in the(**B1**) absence of both AzF and BocK; (**B2**) presence of 1 mM AzF; (**B3**) presence of 5 mM BocK; and (**B4**) presence of both 1 mM AzF and 5 mM BocK.

Experiments were also carried out to demonstrate that the PylRS-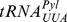 pair does not interfere with suppression of an amber mutation mediated by an evolved *Mj*TyrRS-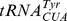 pair. Two plasmids, pETtrio-pylT(UUA)-pylRS-sfGFP134TAG and pEVOL-AzFRS were used to transform *E. coli* BL21(DE3)cells. As shown in **Figure 8A**, growing the transformed cells in four conditions led to different expression levels of sfGFP. When no NAA or only 5 mM BocK was provided in the medium, only a detectable but very low level of sfGFP expression (less than 1 mg/L) was detected. However, addition of 1 mM AzF to the medium supplemented with or without BocK promoted sfGFP overexpression. The ESI-MS analysis of purified sfGFP in all four conditions displayed very interesting spectra (**Figure 8B**). Although a mass peak for sfGFP expressed in two conditions with the supplement of AzF (27,900±1 Da in **Figure 8B** **2**and 27,901±1 Da in **Figure 8B** **4**) matches the calculated molecular weight (27,901 Da) of sfGFP with AzF incorporated at N134, a mass peak for sfGFP expressed in two conditions without the supplement of AzF (27,859±1 Da in **Figure 8B** **1** and 27,860±1 Da in **Figure 8B** **3**) does not match the molecular weight of sfGFP with either Lys/Glu/Gln or BocK incorporated at N134. Instead, this mass peak agrees well with the molecular weight of sfGFP with Phe incorporated at N134 (calculated mass: 27,859 Da).

### AGG Codon Suppression

**Figure 9:**
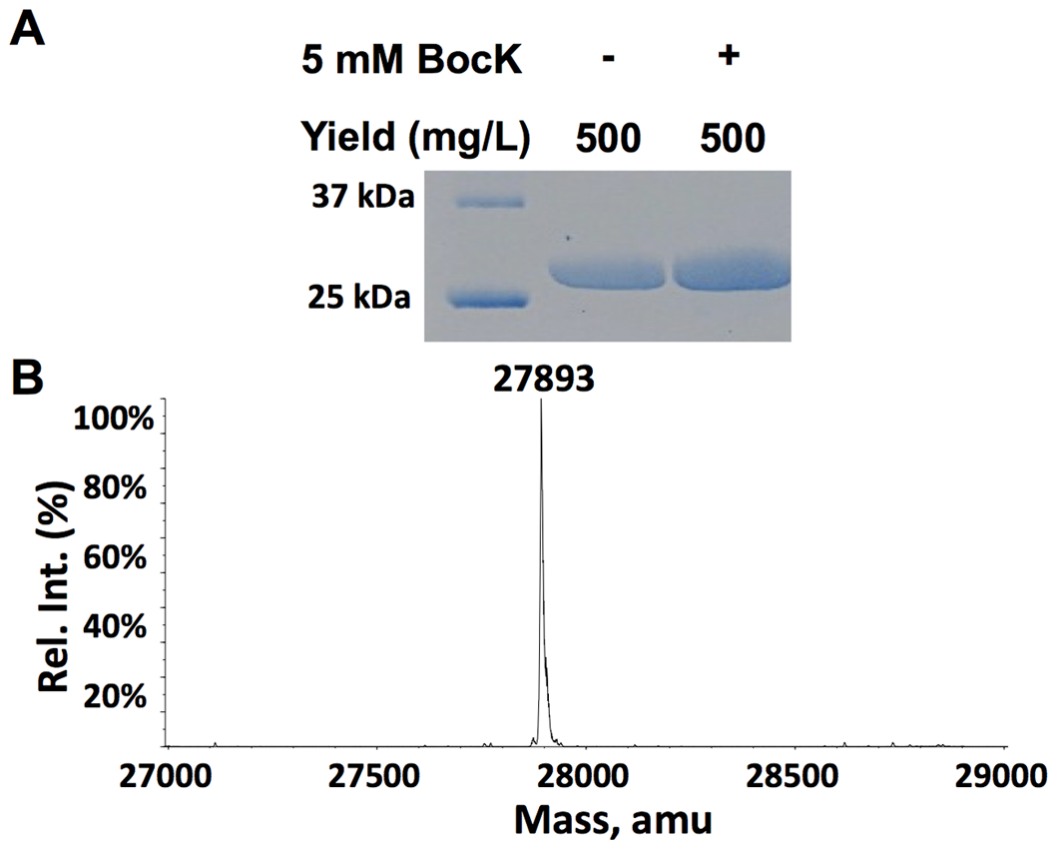
Suppression of an AGG mutation at S2 of sfGFP by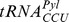. (**A**) Expression of sfGFP in cells transformed with pETtrio-pylT(CCU)-sfGFP2AGG and grown in the absence or presence of 5 mM BocK. (**B**) The ESI-MS analysis of sfGFP expressed in the presence of 5 mM BocK.

*E. coli* BL21(DE3) cells transformed with pETtrio-pylT(CCU)-PylRS-sfGFP2AGG showed similar sfGFP expression levels both in the absence and in the presence of BocK (**Figure 9A**). sfGFP proteins expressed in both conditions displayed one major mass peak at 27,895 Da that agrees well with the theoretical molecular weight of sfGFP with arginine (Arg) incorporated at N134 (calculated mass: 27,896 Da for the full-length protein) (**Figure 9B**).

## Discussion

### Basal Nonsense Suppression in the *E. coli* BL21(DE3) Cell Strain

*E. coli* BL21(DE3) cells transformed with pETtrio-pylT(CUA)-PylRS-sfGFP134TAG and grown in the absence of BocK yielded a sfGFP expression level close to 1 mg/L. The ESI-MS spectrum of the purified sfGFP clearly indicated a Lys, Glu, or Gln residue at the amber mutation site. Since PylRS does not recognize Lys, Glu, and Gln and 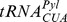
itself does not mediate detectable amber suppression in the *E. coli* Top10 cell strain (data not shown), this low but detectable sfGFP expression level was due to the basal amber suppression in the *E. coli* BL21(DE3) cell strain [6], [27]. This basal amber suppression that was also demonstrated in one separate study from us [27] arises possibly from the recognitions of the UAG codon by its near-cognate tRNAs including 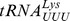/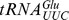/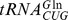 [31], [32].

A similar test with *E. coli* BL21(DE3) cells transformed with pETtrio-pylT(UCA)-PylRS-sfGFP134TGA and grown in the absence of BocK yielded a sfGFP expression level of 8 mg/L. The ESI-MS spectrum of the purified protein showed a Trp residue at N134 of sfGFP. To rule out the possibility that 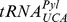
was charged by *E. coli* tryptophanyl-tRNA synthetase (TrpRS) for the delivery of Trp at the designated opal mutation site, we constructed plasmid pETtrio-sfGFP134TGA that did not contain the 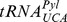 gene for further tests. *E. coli* BL21(DE3) cells transformed with this plasmid yielded a sfGFP expression level of 4 mg/L. The ESI-MS spectrum of the purified protein also showed the incorporation of Trp at N134 of sfGFP. Since we recently demonstrated that 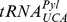 is not recognized by *E. coli* TrpRS, [27] we think sfGFP expressed in the above two conditions resulted from the basal opal suppression in the *E. coli* BL21(DE3) cell strain. The observed different sfGFP expression levels might be caused by different copy numbers of two plasmids in the transformed cells. This basal opal suppression is the consequence of the recognition of the opal codon by tRNA^Trp^. *E. coli* tRNA^Trp^ has a CCA anticodon which is a near-cognate tRNA of UGA. It forms two Watson-Crick base pairs with the 5′ and middle nucleotides of UGA and a wobble base pair with the 3′ nucleotide of UGA, explaining its ability to recognize UGA. We have also excluded the possibility that nucleotide contents around the opal mutation at N134 of sfGFP facilitated the binding of tRNA^Trp^ and induced the Trp incorporation at this site. The *E. coli* BL21(DE3) cells transformed with pETtrio-pylT(UCA)-PylRS-sfGFP2TGA also expressed sfGFP in the absence of BocK and the expressed protein had Trp at the opal mutation site. The sfGFP expression level in *E. coli* BL21(DE3) cells transformed with pylT(UCA)-PylRS-sfGFP2TGA reached to 115 mg/L. This is roughly 20% of the expression level of wild-type sfGFP in *E. coli* BL21(DE3) cells (∼500 mg/L, unpublished data). This high efficiency to read across an opal codon with the binding of a near-cognate tRNA^Trp^ may correlate with the short distance from the opal codon to the start codon.

In contrary to UAG and UGA codons, the UAA codon displays high translation termination stringency in the *E. coli* BL21(DE3) cell strain. Cells transformed with pETtrio-pylT(CUA)-PylRS-sfGFP134TAA showed an undetectable basal ochre suppression level. This can be explained from several aspects. UAG and UGA are recognized by release factor 1 and release factor 2, respectively, whereas UAA is recognized by both release factor 1 and release factor 2. Its recognition by both release factor proteins, in theory, makes the translation termination at UAA more stringent than the other two stop codons. Another reason lies at the nucleotide contents of UAA. Unlike UAG and UGA that could involve a GC base pair interaction, UAA could only form AU pairs or wobble pairs. Its interactions with tRNAs are relatively weak, making its misrecognition less possible than UAG and UGA.

### Amber, Opal, and Ochre Suppression Efficiencies of the PylRS-tRNA^Pyl^ Pairs

When co-expressed with PylRS, all three tRNA^Pyl^ isoforms 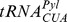,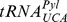, and 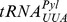 are capable to deliver BocK at their corresponding codon sites. 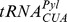 is orthogonal in *E. coli* and displays the highest efficiency in all three isoforms. Given that *E. coli* BL21(DE3) cells transformed with pETtrio-pylT(UUA)-PylRS-sfGFP134TAA did not show a detectable expression level of sfGFP in the absence of BocK, we could conclude that 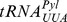 is fully orthogonal in *E. coli*. In comparison to sfGFP expressed in cells transformed with pETtrio-pylT(CUA)-PylRS-sfGFP134TAG and grown in the presence of BocK, the sfGFP expression level in cells transformed with pETtrio-pylT(UUA)-PylRS-sfGFP134TAA and grown in the presence of BocK is five times lower. The low ability of 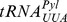 to deliver BocK is possibly due to the relative weak base pair interactions between its UUA anticodon, its UAA stop codon and the availability of both release factor 1 and release factor 2 to stop the translation at a UAA stop codon. In any case, the ochre suppression level achieved by the PylRS-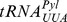 pair is sufficient to promote overexpression of a protein with an ochre mutation. Although a BocK-aminoacylated 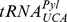 is able to suppress an opal codon for the incorporation of BocK, it does not inhibit the high incorporation of Trp at the same site. As an exogenous tRNA, the sequence and structure of 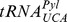 may not be optimal for the protein translation process in *E. coli*. When facing a competition from *E. coli* tRNA^Trp^, the recognition of 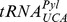 by the *E. coli* translation machinery may be inhibited. Since the 73rd nucleotide serves as a strong recognition element for most tRNAs [33], it was mutated in 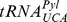 to U, A, and C and searched for a mutant that show a higher opal suppression efficiency. When co-expressed with PylRS in the presence of BocK, 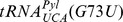
led to higher incorporation level of BocK compared to Trp at the same opal mutation site. However, further mutagenesis with 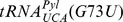
is necessary to fully inhibit the incorporation of Trp at an opal mutation site.

### Anticodon-codon Cross Recognition

The wobble hypothesis was first introduced by Francis Crick in 1966 to explain the observation that a single tRNA is able to efficiently recognize multiple codons [28]. Based on this hypothesis, an ochre suppressor tRNA_UUA_ is also capable of recognizing an amber UAG codon. This is a concern when both UAG and UAA codons are used to code two different NAAs. However, cells transformed with pETtrio-pylT(UUA)-PylRS-sfGFP134TAG and grown in the presence of 5 mM BocK showed a sfGFP expression level close to that from the basal amber suppression. This suggests very weak recognition of UAG by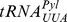. Weak base pairing interactions involved with the UUA anticodon may contribute to the weak recognition of UAG. However, this is certainly not the determining factor since other tRNAs such as 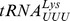
is also involve weak base pairing interactions to recognize multiple codons. One possible explanation for this weak recognition of UAG by 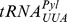 is the tRNA modifications. All cognate tRNAs are known to exhibit similar affinities for the ribosome A site when they bind to corresponding codons [34], [35]. This uniform binding is unexpected as certain codon-anticodon interactions are expected to be more stable than others due to factors such as the GC base pair content. It has been proposed that the specific sequence and post-transcriptional modification status of the tRNA in the region near the anticodon is tuned to ensure nearly indistinguishable binding of tRNAs to the ribosome A site [36]–[38]. This has been the case for tRNAs such as 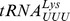
in which both nucleotides at 34 and 37 are post-transcriptionally modified to achieve similar recognitions of AAA and AAG codons [38]. Unlike endogenous tRNAs that have corresponding modification enzymes, 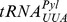 is exogenous and may not be targeted by tRNA modification enzymes in *E. coli*. 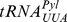
without modifications at its anticodon loop likely has a very weak binding affinity to the ribosome A site to associate UAG. Our finding points out that wobble base pairing at the 3′ nucleotide of a codon is not sufficient for recruiting a tRNA to the ribosome A-site. Additional interactions are required. This aspect needs to be further investigated. Since 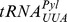
has a low ability to recognize UAG, it is feasible to use an amber suppressor aaRS-tRNA pair and a wild type or evolved PylRS-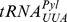 pair to code two different NAAs at amber and ochre mutation sites, respectively, in *E. coli*.

During the anticodon-codon cross recognition analysis to examine whether 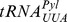 can compete against 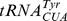 to bind UAG in the ribosome A site, we noticed that Phe was incorporated at N134 of sfGFP which was expressed in cells transformed with pEVOL-AzFRS and pETtrio-pylT(UUA)-PylRS-sfGFP134TAG and grown either in the absence or in the presence of BocK. We think AzFRS can recognize Phe, leading to the misincorporation of Phe. Since AzF is a hydrophobic amino acid and structurally very similar to Phe, one would expect that AzFRS that was originally evolved from *Mj*TyrRS would probably recognize Phe at a relatively low level and charge 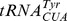 with Phe when AzF is absent in the medium. Although not clearly addressed in existing literature, most evolved *Mj*TyrRS-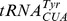 pairs did show significant background amber suppression even in minimal media [39], [40]. Since most evolved *Mj*TyrRS variants are for Phe derivatives, it is highly possible that background amber suppression caused by these evolved *Mj*TyrRS-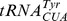 pairs was due to their recognition of either Phe or Tyr or both. In this study, the background amber suppression induced by the AzFRS-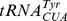 pair inhibited both the basal amber suppression level and amber suppression induced by the PylRS-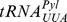 pair. This test also provided evidence that amber suppression mediated by the PylRS-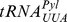 pair is too low to be a concern. It also points out that substrate specificities of evolved NAA-specific aaRSs need to be further characterized.

### AGG Codon Suppression

Our lab and other groups have shown that mutating the anticodon of tRNA^Pyl^ does not significantly affect its interactions with PylRS. Three tRNA^Pyl^ isoforms that are specific for three stop codons are capable to deliver BocK at their corresponding codon sites when co-expressed with PylRS. We were also curious about the ability a tRNA^Pyl^ isoform to suppress a sensing codon. We chose to test on the suppression of the AGG codon since it is a rarely used codon and 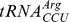 is limitedly expressed in the *E. coli* BL21(DE3) cell strain [41]. However, *E. coli* transformed with pETtrio-pylT(CCU)-PylRS-sfGFP2AGG only expressed sfGFP with Arg at its S2 position in the presence of 5 mM BocK. Increasing the BocK concentration to 10 mM did drive the expression of sfGFP with BocK incorporated at S2 of sfGFP, which was confirmed by the ESI-MS analysis of the purified proteins (data not shown). However, in comparison to the Arg-containing sfGFP isoform that showed a high intensity in the ESI-MS spectrum of the purified sfGFP, the intensity for the BocK-containing sfGFP isoform was very low. This indicates 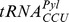 is charged by PylRS with BocK in *E. coli* and is able to deliver BocK at an AGG codon site. However, the PylRS- 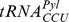 pair cannot compete efficiently against the endogenous Arg incorporation system at the AGG codon. Similar to 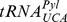, the sequence and structure of 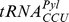 may not be optimized for the protein translation machinery in *E. coli*.

In summary, the suppression efficiencies of the original and three tRNA^Pyl^ variant, the cross recognition between nonsense codons and tRNA^Pyl^ anticodons in the *E. coli* BL21(DE3) cell strain have been investigated. Among all tRNA^Pyl^ isoforms, 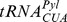
has the highest suppression efficiency for the delivery of BocK at its corresponding codon. Besides its orthogonal nature in *E. coli*, 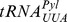
does not induce a significant level of suppression at an amber codon. This is contrary to the wobble hypothesis and makes it feasible to use amber suppressing aaRS-tRNA pair and the PylRS-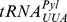 pair to code two different NAAs at amber and ochre codons respectively in *E. coli*. Our study also demonstrates the PylRS-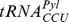 pair cannot efficiently deliver BocK at an AGG codon site. Further work to optimize the sequence and structure of 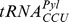 for the *E. coli* translation machinery may be necessary to increase the BocK incorporation efficiency and suppress the Arg incorporation at the AGG codon.
